# Innate resistance of PD-1 blockade through loss of function mutations in JAK resulting in inability to express PD-L1 upon interferon exposure

**DOI:** 10.1186/2051-1426-3-S2-P311

**Published:** 2015-11-04

**Authors:** Daniel Shin, Angel Garcia-Diaz, Jesse Zaretsky, Helena Escuin-Ordinas, Siwen Hu-Lieskovan, Nicolaos J Palaskas, Willy Hugo, Marie Sara Komenan, Bartosz Chmielowski, Grace Cherry, Beata Berent-Maoz, Thomas G Graeber, Roger Lo, Begonya Comin-Anduix, Antoni Ribas

**Affiliations:** 1 UCLA, Los Angeles, CA, USA; 2 UCLA Molecular and Medical Pharmacology, Los Angeles, CA, USA; 3 David Geffen School of Medicine at UCLA, Los Angeles, CA, USA; 4 Division of Hematology - Medical Oncology, UCLA Jonsson Comprehensive Cancer Center, Los Angeles, CA, USA; 5 UCLA, School of Medicine, LA, CA, USA; 6 University of California at Los Angeles Medical Center, Los Angeles, CA, USA

## Introduction

PD-L1-negative tumors assessed by immunohistochemistry often still respond to PD-1 blockade. PD-L1 is inducible by interferon, therefore, absolute negative tumors are the ones unable to up-regulate PD-L1 in response to interferons. Genetic mutations in the interferon receptor signaling pathway leading to loss of PD-L1 up-regulation were hypothesized to exhibit innate resistance to PD-1 blockade.

## Experimental procedures

After optimization, 50 primary human melanoma cell lines were exposed to interferons (alpha, beta and gamma) and PD-L1 expression was measured. Interferon signaling was assessed by single cell phospho-proteomics, pSTAT1 (Y701), pSTAT3, pSTAT5, pSTAT6 expression level by flow cytometry (FACS LSRII). Western blot assessed JAK1/JAK2/IRF1 as well as STAT/pSTAT expression and Whole exome sequencing was performed by next generation sequencing for three selected melanoma cell lines and on biopsies from 25 patients with advanced melanoma treated with anti-PD-1 therapy.

## Results

Three out of 50 melanoma cell lines were unable to up-regulate PD-L1 in response to interferon gamma; two of them had disruptive mutations in JAK1 or JAK2, and a third one had a defect in expression of IRF1 in response to interferons. Western blot analysis confirmed loss of function for the JAK1/JAK2 mutations and loss of downstream IRF1/STAT1/3/5 phosphorylation events. Whole exome sequencing of biopsies from 15 patients with metastatic melanoma who had objective response to PD-1 blockade (pembrolizumab) showed no homozygous inactivating mutations in interferon signaling pathway genes. Interestingly, one patient with the highest mutational load out of 10 patients without clinical response to PD-1 blockade had an amplified allele of JAK1 with a P429S mutation in the src-homology (SH2) domain. A cell line derived from this patient showed lack of sustained up-regulating of PD-L1 expression in response to interferon gamma by, and Western blot confirmed loss of JAK1 expression. Immunohistochemistry of tumor biopsy for this patient showed few CD8+ T cells.

## Conclusions

This study has defined genetic mechanisms of innate resistance to PD-1 blockade which lead to inhibition of adaptive PD-L1 expression in patients with advanced melanoma. This work suggests lack of interferon-gamma induced PD-L1 upregulation has the potential to be a negative selective marker for PD-1 blockade therapy.

